# Cervical Cancer Screening in Women Living With HIV in Suriname

**DOI:** 10.7759/cureus.105234

**Published:** 2026-03-14

**Authors:** Lycke Woittiez, Andjanie Soekdew, Els Dams, Kees Brinkman, Jimmy Roosblad, Michèle van Vugt, Stephen G Vreden

**Affiliations:** 1 Internal Medicine, Academic Hospital Paramaribo, Paramaribo, SUR; 2 Faculty of Medicine, University of Amsterdam, Amsterdam, NLD; 3 Internal Medicine, Diakonessenhuis, Paramaribo, SUR; 4 Internal Medicine, Onze Lieve Vrouwen Gasthuis, Amsterdam, NLD; 5 Clinical Chemistry, Academic Hospital Paramaribo, Paramaribo, SUR; 6 Infectious Diseases, Amsterdam University Medical Center, Amsterdam, NLD; 7 Internal Medicine, Foundation for the Advancement of Scientific Research in Suriname, Paramaribo, Suriname

**Keywords:** cervical cancer, hiv, screening, suriname, wlwh

## Abstract

Background

Suriname has an estimated human immunodeficiency virus (HIV) prevalence of 1.6% among adults aged 15 to 49 years. Women living with HIV (WLWH) have a higher risk of developing cervical cancer or dysplasia. The World Health Organization (WHO) guidelines advise cervical screening once every three years, starting at age 25, when using visual inspection with acetic acid (VIA).

Objective

The primary objective of this study was to determine how often WLWH in Suriname undergo cervical screening. The secondary objective was to explore factors associated with cervical screening participation.

Methodology

In this single-center study, data were collected from all WLWH visiting the HIV outpatient clinic of the Academic Hospital Paramaribo during six weeks in 2023. The last cervical screening date was registered. To explore factors associated with the cervical screening rate, these women were asked to fill out a questionnaire.

Results

During the study period, 195 WLWH visited the outpatient department. Of these, 134 (68.7%) had ever gone for cervical screening, and 77 (39.5%) had gone for cervical screening in the past three years.

A total of 134 WLWH participated in the questionnaire. Of those women, 99 (73.9%) were not aware of the higher risk of cervical cancer in WLWH.

Conclusions

Although a majority of WLWH had done cervical screening at least once in their lives, less than 40% were screened in the last three years in accordance with WHO guidelines. Strategies to increase the number of women going for cervical cancer screening should be explored in the coming years.

## Introduction

Human immunodeficiency virus (HIV) is a major global health issue [1]. Between 2015 and 2020, the HIV prevalence in Suriname ranged from 1.1% to 1.3%, while the 2023 data report an HIV prevalence of 1.6% among adults aged 15 to 49 years [2].

HIV is associated with reduced clearance of HPV infection, leading to an increased likelihood of developing high-grade cervical intraepithelial neoplasia (CIN III), carcinoma in situ (CIS), and (micro) invasive carcinoma [3]. Women living with HIV (WLWH) have a sixfold higher risk of developing cervical cancer [4,5], and the risk of HPV infection progressing to dysplasia or cancer is higher in women who are not on combination antiretroviral therapy (cART) [6]. Starting cART improves HPV clearance and may cause regression of histological abnormalities [6,7]. 

Because of the increased risk of cervical (pre)cancer, it is important to regularly screen WLWH for cervical cancer. The World Health Organization (WHO) recommends cervical screening for WLWH aged 25 to 50 years: every three years using cytology or visual inspection of the cervix with acetic acid (VIA), or every three to five years using human papillomavirus (HPV) DNA testing [8].

Suriname is a country located in South America, with a multi-ethnic population of approximately 600,000 people, consisting of Hindustani, Maroon, Creole, Javanese, indigenous, and other ethnicities [9,10]. Although Suriname is classified by the World Bank as an upper-middle-income country, the ongoing economic crisis has substantially affected healthcare resources and access [11]. There is no formal cervical cancer screening program in place, but the Surinamese guideline advises starting yearly cervical cancer screening from age 23 [12]. The estimated screening coverage is less than 30% [13]. For diagnosis, either a Pap Smear or a VIA is done. If the result is abnormal, colposcopy and biopsy are performed.

The extent to which WLWH in Suriname are regularly screened for cervical cancer is unknown. The primary objective of this study was to analyze the rate and frequency of screening for cervical cancer in WLWH. The secondary objective was to explore factors associated with attending cervical cancer screening.

## Materials and methods

Study design and setting

We conducted an observational study at the outpatient department of the Academic Hospital Paramaribo (AZP). This is the largest hospital in Suriname. At the end of 2023, 1870 people were living with HIV (PLWH) registered in the outpatient department of the AZP. This is approximately half of the estimated 3900 diagnosed PLWH in Suriname in 2023 [2].

Data were collected between 5 September 2023 and 13 October 2023. Since this was an exploratory study, no a priori sample size calculation was performed. The sample size was determined by the number of eligible women attending during the predefined six-week data collection period for reasons of convenience.

Patient recruitment

WLWH attending the HIV outpatient clinic at the Academic Hospital between September 5 and October 13, 2023, were invited to complete a questionnaire on their cervical screening history.

Inclusion and exclusion criteria

All women aged 25-65 years who had been living with HIV for at least six months and who attended the HIV outpatient clinic during the study period were included. Women who did not meet these criteria were excluded, and no additional exclusion criteria were applied.

Only the eligible women who provided consent were included in the questionnaire component of the study. 

Data collection

A trained researcher was available to support participants in completing the questionnaire. In the questionnaire, women were asked about age, ethnicity, education, and duration of HIV infection; we also recorded treatment and laboratory results. The full questionnaire is available in the Appendix. Furthermore, WLWH were asked whether they were aware of their increased risk of cervical cancer and when they were last screened. Those who had never been screened were asked to provide the reason. Screening history was based solely on self-reported data.

After completing the questionnaire, women were informed about cervical screening and its importance. If a patient’s last cervical screening was performed more than three years ago, the patient received a referral for cervical screening. Filling out the questionnaire was voluntary, and if patients declined to participate, only the reported date of the last cervical examination was recorded. For all participants, the last viral load within six months before the current visit was recorded, if available. Viral load values <100 copies/mL were considered undetectable based on the threshold of the local in-house laboratory assay. The location of residence was recorded using the electronic patient registration system and then classified as *urban coastal*, *rural coastal*, or *rural interior*. 

All data were collected using Castor (Castor Electronic Data Capture, Amsterdam, The Netherlands). A separate patient identification file was created.

Outcomes

The primary outcome of this study was the proportion of WLWH attending the Academic Hospital who had undergone cervical screening less than three years ago. Although national guidelines recommend annual screening from the age of 23 years, a three-year interval based on WHO recommendations was used to define recent screening in order to allow international comparability. The secondary outcomes were the percentage of WLWH who were ever screened for cervical cancer and descriptive exploration of factors associated with cervical screening attendance. 

Statistical analysis

Categorical data were reported as frequencies and percentages, and continuous data were reported as mean and standard deviation (SD) or median and interquartile range (IQR), depending on the distribution of the data. For statistical analysis, SPSS version 25 (IBM Corp., Armonk, NY) was used.

Ethical considerations

The study protocol was reviewed and approved by the Institutional Review Board of the Academic Hospital Paramaribo, Suriname (letter number 362; approval date: September 4, 2023). The IRB approved the use of verbal informed consent, and all participants provided verbal informed consent before completing the questionnaire.

## Results

Participants

During the inclusion period, 195 WLWH visited the HIV clinic. Of these, 134 (68.7%) agreed to complete the questionnaire.

Patient characteristics

Characteristics of the 195 WLWH who visited the outpatient department during the study period are shown in Table 1. The median age was 44.0 years (IQR 39.0-53.0), and the majority resided in urban areas. Overall, 134 women (68.7%) had undergone cervical cancer screening at least once in their lifetime, and 77 (39.5%) had been screened in the past three years. Most women (156, 80.0%) had an undetectable viral load.

**Table 1:** Data of 195 women living with HIV (WLWH) attending the HIV clinic during the study period. IQR, interquartile range

Characteristics			
Median age - year (IQR)		44.0	14.0
Last viral load			
	Undetectable (<100 copies/mL), *n* (%)	156	80.0%
	Detectable, *n* (%)	39	20.0%
Place of residence, *n* (%)			
	Urban	169	86.7%
	Rural coastal	16	8.2%
	Rural interior	9	4.6%
	Unknown	1	0.5%
Time since last cervical screening, *n* (%)			
	<3 years	77	39.5%
	≥3 years	57	29.2%
	Never	61	31.3%

Cervical screening in WLWH

A subset of 134 women was interviewed to explore potential reasons for not attending cervical cancer screening. Details about these interviewed women are shown in Table 2. The median age was 44.0 years (IQR 38.0-52.3), and the majority (100, 74.6%) had an undetectable viral load.

**Table 2:** Data of 134 women living with HIV (WLWH) who participated in the questionnaire. cART, combination antiretroviral therapy

			Frequency	Mean (SD) or median (IQR) or %
Median age - year (IQR)			44.0	38.0-52.3
Ethnicity - *n* (%)				
	Creole	15.7% of population	47	35.1%
	Hindustani	27.4% of population	16	11.9%
	Javanese	13.7% of population	1	0.7%
	Maroon	21.7% of population	32	23.9%
	Other	21.0% of population	38	28.4%
	Unknown	0.6% of population	0	0%
Highest completed education – *n* (%)				
	Did not finish primary education		25	18.7%
	Finished primary education		41	30.6%
	Finished secondary or higher education		68	50.7%
Years since HIV diagnosis – mean (SD)			10.5	5.9
Years on cART – mean (SD)			8.6	5.4
Latest HIV viral load (<6 months before visit)				
	Undetectable (<100 copies/mL)		100	74.6%
	Detectable (>100 copies/mL)		34	25.4%
Time since last cervical screening, *n* (%)				
	<3 years		45	33.6%
	≥3 years		40	29.9%
	Never		49	36.6%
Aware of higher risk cervical cancer in WLWH? – *n* (%)				
	Aware		20	14.9%
	Not aware		99	73.9%
	Unknown		15	11.2%
Did the doctor mention cervical cancer screening? – *n* (%)				
	Yes		11	8.2%
	No		51	38.1%
	Unknown		72	53.7%

Of these 134 women, 85 (63.4%) underwent cervical screening at least once in their lives, and 45 (33.6%) were screened in the last three years. The majority of women (99, 73.9%) mentioned they were not aware of the higher risk for cervical cancer in WLWH. Only 11 (8.2%) confirmed that the doctor discussed cervical cancer screening with them.

Of the 49 women who had never done cervical screening, 15 (30.6%) had never heard of cervical screening before. Of the 34 women who knew they should go for cervical screening, different reasons for not going were given. Sixteen women (47.1%) did not know what the investigation was for, and seven (20.6%) did not think it was useful. Seven women (20.6%) feared the investigation or its results. Four women (11.8%) did not know how to arrange the logistics, three (8.8%) said the doctor never mentioned the need for screening, and two women (5.9%) did not have time. Three women (8.8%) mentioned other reasons. None of the women mentioned costs or transport problems as the main reason for not going for cervical screening.

## Discussion

The primary goal of this study was to determine the rate and frequency of cervical screening among WLWH. Although only 77 (39.5%) of the WLWH had been screened for cervical cancer in the last three years, 134 (68.7%) had been screened at least once in their lifetime. The target in the WHO global strategy to eliminate cervical cancer is that 70% of women are screened for cervical cancer before the age of 45 [14]. Although we do not know how many women were screened before age 45, our overall screening rate is close to this 70% target.

Our observed lifetime screening prevalence of 68.7% lies between estimates reported for cervical screening among women living with HIV (WLWH) in lower-middle-income countries (LMICs) of 30.2% and in high-income countries (HICs) of 92.4% [15]. This intermediate level may reflect Suriname’s UMIC status. Furthermore, it could be due to a large proportion of women in this study residing in urban areas, with better access to cervical screening facilities than those living in rural areas. Still, our reported screening rates in WLWH are insufficient in the context of their risk of cervical pathology. Therefore, we should aim to increase the screening rate.

The secondary objective of this study was to explore factors associated with attending cervical screening. In our study, most women reported they were not aware of the higher risk of cervical cancer in WLWH. Furthermore, they mentioned they did not know what the screening was for, or thought it was not useful. This shows that lack of information and knowledge were the main reasons for not going for screening.

Other studies looking into this subject showed similar results.

Knowledge about cervical screening was also found to be an important factor in a review from sub-Saharan Africa. The most important barriers identified in that study were knowledge about cervical cancer and screening, risk perception, fear of the test and its result, access to and cost of testing services, and partner attitude and acceptance [16].

Another review from 2020 examining barriers to cervical cancer screening identified lack of knowledge, stigma, fear, and absence of symptoms as the most important factors; lack of financial resources and lack of partner acceptance were also reported [17].

A systematic review from 2023 showed that in LMICs, women living in urban settings and those of older age and with higher education levels were more likely to attend screening [15]. Our study was not sufficiently powered to examine these factors.

All these studies confirm our finding that lack of information and knowledge are the main reasons for not attending screening. Whether the other factors, such as finance and the attitude of the partner, play a significant role cannot be determined by our study.

Practical implications

Several potential barriers to cervical cancer screening were found in our study. Many women reported that they did not know they should attend screening regularly, it was not offered, they forgot, or they did not think it was important.

These are all factors that could potentially be addressed by creating more awareness and explaining more about this subject during the outpatient visits of WLWH. This means there is an important role for HIV clinicians, and that they should include information about cervical screening in every consultation.

A Cochrane review investigating options to encourage the uptake of cervical cancer screening in women showed that (personal) invitations, educational material, the involvement of lay health care workers, and HPV self-testing could all improve the uptake of screening [18]. However, there are few data about ways to improve cervical cancer screening specifically in WLWH. A review from 2017 identified models to integrate HIV and cervical cancer services. This could be done by teaching the staff in the HIV outpatient department visual inspection techniques, or implementing same-day referral to the gynecology department [19]. Another option could be to implement HPV self-testing [20].

Since the lack of awareness of the importance of cervical cancer screening seems to be a major factor influencing the cervical screening rate, systematically providing patients with information about the importance of this procedure should be the first step in improving cervical screening rates.

Strengths and limitations of this study

The most important strength of this study is that it is the first study to describe cervical screening rates in WLWH in Suriname and, as far as we know, in the region. Another strength is the presence of a trained investigator to help women fill out the questionnaire. Although only one hospital was included, approximately 50% of diagnosed PLWH in Suriname are attending this clinic, meaning the study sample is likely representative.

A limitation of this study is the short inclusion period and the relatively small sample size. Because only women attending the clinic during the inclusion period were included, women who visit the outpatient department less regularly may have been underrepresented. Although some women previously lost to follow-up re-attended during this period and were therefore captured, selection bias cannot be excluded. This may have resulted in an overestimation of the reported screening rate. Another possible limitation is that data were collected in the presence of an interviewer, potentially leading to bias due to socially desirable answers. Furthermore, a non-validated questionnaire was used, and cervical cancer screening history was based solely on self-reported data.

Results of cervical cancer screening were not available in this study. However, for our research question, namely whether women with HIV regularly go for their cervical screening, the result of the screening is not relevant.

## Conclusions

The results of this study indicate that approximately two-thirds of included WLWH attending the outpatient department of the largest HIV clinic in Suriname during the inclusion period had ever been screened for cervical cancer. However, less than half of the WLWH had been screened within the past three years, in line with WHO recommendations.

Targeted efforts are needed to increase the cervical cancer screening rate. Interventions such as providing educational materials and, in the future, implementing HPV self-testing could contribute to improving screening coverage.

## Appendices

Appendix 

**Table 3:** Questionnaire.

Question	Answer
What is your age?	
What is your ethnicity?	Creole Hindustani Maroon Indigenous Chinese Javanese Mixed White Unknown Other, namely
What is your highest completed education?	None Elementary school Mulo Natin Havo VWO HBO-bachelor HBO-master WO-bachelor WO-master Other, namely
How many years have you been living with HIV?	Either a number or unknown.
When did you start with ART?	Either a number or unknown.
Was the last viral load measurement undetectable?	Yes No Unknown
Were you aware that women with HIV have a higher risk of developing cervical cancer?	Yes No
Have you undergone a cervical screening in your life?	Yes No Unknown
Has your physician at AZP ever mentioned that you should undergo a cervical screening or that you have a higher risk of attaining cervical cancer?	Yes No Unknown
If the answer to question 9 was no: 9.1 Have you ever heard of a cervical screening?	Yes No
If the answer to question 9.1 was yes: 9.2 What are the reasons why you never have gone?	I don’t know what the screening is for I dread the examination. I dread the result of the screening. Cost issues Travel issues Problems with my general practitioner It does not seem useful to me. Other, namely
If the answer to question 9 was “yes”: 9.3 Have you ever been to Stichting Lobi Health Center for cervical screening?	Yes No Unknown
If the answer to question 9 was “yes”: 9.3 Have you ever been to a gynecologist for cervical screening?	Yes No Unknown
If either question 9.2 or 9.3 was equal to “yes”: 9.4 Do you regularly go for a cervical screening?	Yes No Unknown
If the answer to question 9.4 was equal to either “no” or unknown: 9.5 What are the reasons for not going regularly?	I forgot. I did not know it was that important to do. The physician never talked about it. It doesn’t seem useful to me. Cost problems. Transportation problems. Fear. Problems with the general practitioner. The examination is uncomfortable. Unknown. Other, namely.
If the answer to question 9 was yes: 9.5 When was the last time you did a cervical screening?	0-1 years ago 1-2 years ago 2-3 years ago 3-5 years ago More than 5 years ago Unknown
If the answer to question 9 was yes: 9.6 Have you received the results of your latest cervical screening?	Yes No Unknown
If the answer to question 9.6 was no: 9.7 Why did you not receive your latest result?	Fear Cost/transportation problems Problems with the GP Forgotten Unknown Other, namely
If the answer to question 9.6 was “yes”: 9.8 What were the results of the latest cervical screening?	Abnormalities No abnormalities Unknown
What insurance do you have?	SZF Bazo (SZF) Self Reliance Assuria Bedrijven ER (Eigen rekening) Parsasco
Have you ever had cervical abnormalities?	Yes No Unknown
Does cervical cancer occur in your family?	Yes No Unknown
